# The efficacy of AuNP-probe conjugate nanobiosensor in non-amplification and amplification forms for the diagnosis of leishmaniasis

**DOI:** 10.1186/s12879-022-07835-z

**Published:** 2022-11-12

**Authors:** Someye Deris, Mahmoud Osanloo, Abdolmajid Ghasemian, Saeed Ataei, Maryam Kohansal, Sahar Samsami, Ava Yazdanpanah, Alireza Ebrahimnezhad, Ali Ghanbariasad

**Affiliations:** 1 Student Research Committee, Fasa University of Medical Sciences, Fasa, Iran; 2 Department of Medical Nanotechnology, School of Advanced Technologies in Medicine, Fasa University of Medical Sciences, Fasa, Iran; 3 Noncommunicable Diseases Research Center, Fasa University of Medical Sciences, Fasa, Iran; 4 Biotechnology Research Center, Shiraz University of Medical Sciences, Shiraz, Iran; 5 Department of Medical Biotechnology, School of Advanced Technologies in Medicine, Fasa University of Medical Sciences, Fasa, Iran; 6 Department of Medical Nanotechnology, School of Advanced Medical Sciences and Technologies, Shiraz University of Medical Sciences, Unit 23, 2nd Floor, Almass Building, Alley 29, Ghasrodasht St., Shiraz, Iran

**Keywords:** Gold nanoparticles, *Leishmania* spp, Diagnosis, Biosensor, Hybridization

## Abstract

Nanobiosensor platforms have emerged as convenient and promising approaches with remarkable efficacy for the diagnosis of infectious diseases. Gold nanoparticles (AuNPs) have been widely used due to numerous advantageous properties such as optical, electrical, physicochemical and great biomolecules binding capabilities. This study aimed to apply AuNP-Probe Conjugate for the detection of *Leishmania* spp., using colorimetric and amplification methods targeting parasitic ITS2 fragment. The first method was carried out by hybridization of 10µL of DNA with 4 µL of probe and addition of 5 µL of 0.2 N HCl (non-amplification method). Second method was followed by polymerase chain reaction (PCR) amplification using thiolated primer, 5 µL of AuNP and 5 µL of 0.2 N HCl. The appearance of red and purple colors indicated positive and negative results, respectively. The minimum of detection for non-amplification and amplification methods for three strains of *Leishmania* namely *L. major, L. tropica* and *L. infantum* were determined to be 32 fg/µL and 16 fg/µL, respectively. Sensitivity for detection of visceral leishmaniasis (VL) for non-amplification and amplification methods included 96% and 100%, respectively and for cutaneous leishmaniasis (CL) included 98% and 100%, respectively. The results of this investigation revealed that sensitivity of amplification method was the same as RT-qPCR, while that of non-amplification method was lower. However, this method was promising because of no need for any equipment, high specificity, enough sensitivity, low cost and rapidity (less than 30 min) to complete after genomic DNA extraction.

## Introduction

Leishmaniasis is a prevalent vector-borne infection which is caused by 21 zoonotic *Leishmania* spp in more than 90 countries [1, 2]. According to the world health organization (WHO), about one million patients are being victimized every year by three forms of leishmaniasis. Parasites are transmitted by different sandflies genera worldwide as *Phlebotomine*, *Lutzomyia* and *Nissomya* genera [3, 4]. Leishmaniasis has three forms of clinical manifestations namely VL, CL and mucocutaneous leishmaniasis, which depend on the types of phagocytic cells involved during the infection [1, 5, 6]. Thus far, several approaches have been used for *Leishmania* spp., detection and monitoring patients. Microscopic examination and isolation of the parasites are the conventional method of diagnosing of the disease; however, microscopic observation is an arduous task which depends on the skillfulness of the staff. Additionally, parasites isolation requires their cultivation is a time-consuming approach with low sensitivity [7]. Although immunoassay tests have also been used to detect leishmaniasis such as enzyme linked immune assay (ELISA), the tests are subject to cross-reactivity with other agents namely *Trypanosoma* spp., and *Plasmodium* spp., species, lowering their specificity. Over the last decade, molecular-based approaches such as polymerase chain reaction (PCR) and real-time PCR have been employed for the rapid, accurate and sensitive diagnostic purposes as they have enabled researchers to focus on specific genetic loci which were unique to the pathogen under investigation [8]. Although most molecular diagnostic approaches have great sensitivity and specificity, fast performance and low contamination risk, these methods require skilled personnel, specialized reagents and expensive high-tech facilities which is a significant downside to their application particularly in low-income countries [9]. To overcome these problems, over the past decades Nanoparticle (NP)-based diagnostic approaches have provided highly attractive [4, 10], easy to perform, precise and efficient approaches for sensing and detecting small amounts of biological material, such as DNA, without the need for amplifying the genome [11]. NP-based colorimetric methods are broadly used as an ultrasensitive molecular recognition approaches. Gold NPs (AuNPs) have provided novel alternative approach in the field of diagnostics owing to their unique optical, electric and physicochemical features along with containing a variety of functional chemical groups [12]. In terms of detection, NPs offer several straight forward approaches such as change of color upon aggregation via induction with acidic solutions [13, 14]. To this end, here we aimed to design and construct an AuNP-conjugated probe as a Geno-sensor to capture the ITS2 (Internal transcribed spacer) of *Leishmania* species to visually detect leishmaniasis utilizing two approaches. In the first approach, we strived to capture the target DNA with AuNP probe. However, in the second approach we strived to PCR amplify the target DNA, while it was captured by the AuNP-probes conjugate. The results of the two approaches were comparatively analyzed. Furthermore, the quantitative real-time PCR (RT-qPCR) technique was exploited as the reference method of detection.

## Materials and methods

### Sample preparation

Each *L. major, L. tropica and L. infantum* standard strain was cultured in the Roswell Park Memorial Institute (RPMI) medium supplemented with 15% fetal bovine serum (FBS), 100 IU of penicillin and 100 μg/mL of streptomycin at 25 °C in a shaker incubator. Positive controls were provided via cultivating the standard strains of *L. major* (MHOM/IR/75/ER)*, L. tropica* (MHOM/SU/74/K27) and *L. infantum* (MCAN/IR/97/LON490), which were obtained from Pasteur Institute of Iran. The *Toxoplasma gondii, Escherichia coli, cryptosporidium parvum and Candida albicans* served as a negative control species.

### Genomic DNA isolation

DNA extraction was performed according to the manufacture’s protocol using FlexiGene DNA kit (Qiagen). The isolated DNA was stored at − 20 °C. The concentration of the DNA was determined by measuring the optical density at 260 nm using a Nanodrop Spectrophotometer (Biotech, Synergy HTX).

### Synthesis and characterization of AuNPs

Citrate-capped AuNPs with an average diameter of about 20 nm were synthesized using previous method described by [15, 16]. Briefly, a 50 mL solution containing 0.7 mM of HAuCl_4_ was prepared and heated under reflux. Once boiled, 5 mL of 25.9 mM trisodium citrate were added under vigorous stirring which was continued for 30 min for color change to deep red indicating the formation of AuNPs. Then the solution was left to reach at room temperature. According to Beer’s law and the extinction coefficient of 20 nm AuNPs at 524 nm wavelength, the particle concentration was estimated to be 10 nM. The AuNPs solution was stored at 4 °C in a dark environment for further use.

The mean diameter values of particle size (PS) and particle size distribution (SPAN) of the AuNPs were determined using dynamic light scattering (DLS) (scatteroscope, K-ONE NANO. LTD, Korea). Morphology, size, and the characterization of the particles were investigated using Transmission Electron Microscope (TEM) (Libra 120 TEM) by means of accelerating voltage of 400 kV. The optical properties of gold dispersions were investigated using UV–Vis spectrophotometer (UVIKON 923 UV–Vis spectrophotometer).

### Probe design

The ITS2 sequence was selected to serve as a template. A 20-mer single-stranded oligonucleotide was selected and thiolated at the 5′ end to serve as probe which was ordered from the Bioneer Company (South Korea). The 5′ thiol group facilitated conjugation with colloidal AuNPs (5′-SH-AGGCGTGTGTTTGTGTTGTG—3ʹ) [17, 18]. The probe is designed to specifically detect *L. tropica*, *L. major* and *L. infantum*. To do this, Basic Local Alignment Search Tool (BLAST) of the National Center for Biotechnology Information (NCBI) was exploited. NCBI database was used for primer BLAST and MEGA6 confirmed the primers specifically for the detection of *Leishmania* species.

### Conjugation of AuNPs nanoparticles with the oligonucleotide probes

The thiolated probe was initially suspended in 20 µL TCEP (0.2 M) at room temperature for 1 h to reduce and activate the thiol groups and then conjugation with AuNPs was carried out (26). To remove TCEP, after adding 5 µL sodium acetate (3 M) and 200 µL cold absolute ethanol, the mixture was incubated at − 20 °C for an hour. Once the solution was centrifuged at 18,000 *g* for 15 min, the supernatant was decanted and the pellet was left to dry completely at ambient temperature. 1 mL of AuNPs was centrifuged and pelleted at 16200 *g* for 15 min at 4 °C and then suspended in another 1 mL AuNPs suspension. The AuNPs were mixed with 20 µL of thiolated oligonucleotide probe (Concentration) and left at ambient temperature with shaking for an overnight. Next, 100 µL phosphate buffer (100 mM, pH:7) and 11 µL sodium dodecyl sulfate (SDS) (10%) were added to the mixture of probe and NPs and placed in shaker for 30 min. Next, every 8 h (for seven times), the AuNPs-probe mixture was supplemented with a high-salt solution buffer (NaCl 2 M, Na_2_PO_4_ 10 mM). The first time 4.4 µL and for the next subsequent times 8.8 µL of high-salt solution was added. The solution was centrifuged at 16,000 *g* for 30 s at 4 °C. After decanting the supernatant, the mixture was washed with 500 µL wash buffer, (NaCl 1.5 Mm, 0.1% SDS in PBS Buffer) and centrifuged at 16,000 *g* at 4 °C for 30 s. The supernatant was decanted and the pellet containing AuNP-probe conjugate was re-suspended with 200 µL wash buffer and stored at 4 °C while wrapped around aluminum foil until further use. To confirm the conjugation process, salt-induced AuNP probe aggregation test was performed. To do so, 1 µL of MgCl_2_ (5 mM) was added to 5 µL of AuNP-probe conjugate and non-conjugated nanoparticle separately. After 10 min of incubation, the color change was analyzed. Proper conjugation should not induce any color change, while non-conjugated NPs precipitate changes the color of the solution.

### Non-amplification colorimetric assay

In this method, colorimetric assay was employed. Briefly, the target DNA (10 µL) was denatured at 95 °C for 5 min and was immediately ice cooled. Once the separated DNA strands were stabled at low temperature, the conjugated NPs (4 µL) was added and thoroughly mixed with the DNA. After incubation at 63 °C for 10 min, the hybridization was halted via the addition of HCl (5 µL, 0.2 M). Finally, the mixture was incubated for 5 min before visual and spectrophotometric (400–700 nm) analysis of the result. As for the negative controls, samples lacking the target DNA were subjected to the same process.

### Amplification colorimetric assay

Herein, the AuNP-probe conjugates were used to capture the target DNA and subjected to PCR amplification in which the probe served as forward primer, and 5′-ACAAAGGTTGTCGGGGGTG-3′ as reverse primer amplifying a 517 bp length product. A unique PCR Master Mix (Taq OptiMix CLEAR Master Mix, Amplicon, Denmark) was used. Nanoparticle aggregation was analyzed following the addition of HCl. In this method, 5 µL of PCR products was supplemented with 20 µL of NPs and 2.5 µL of HCl (0.2 M).

### RT-qPCR technique

Primers were designed by Allele ID software and checked by primer3 of NCBI based on the ITS2 region (Forward: AGGCGTGTGTTTGTGTTGTG Reverse: GCAAGCACCAGAGAGGAGTT). the reaction mixture consisted of 7.5 μL of High Rox Red master mix (Amplicon, Denmark), 0.5 μL of 10 μM of forward and reverse primers, and 1 μL of DNA template. The program (Absent- present) was performed on the (ABI Real-time PCR, Sep one plus system) under the condition: early denaturation 95 °C for 3 min, denaturation step 95 °C for 45 s, annealing 55 °C for 45 s, extension 72 °C for the 60 s and final extension 72 °C for 10 min. The specificity of RT-qPCR was achieved through a Melt curve which included *Leishmania* species serial dilution. The melting diagram of dilutions was evaluated based on the location of the peak.

### Determination of the threshold of detection

**Fig. 1:**
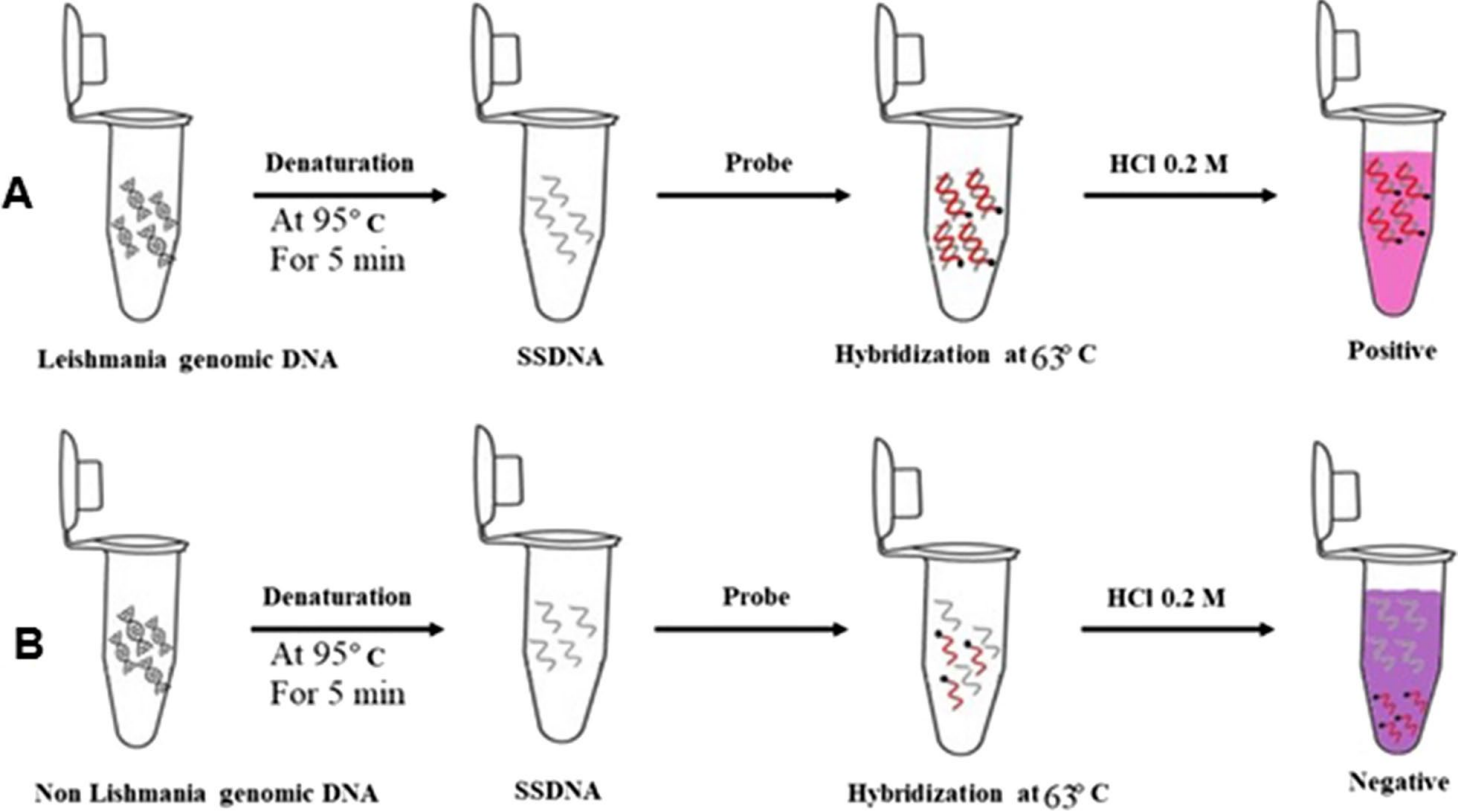
Schematic illustration of the process; **A** dispersal of hybridized AuNPs-probes in the solution and **B** AuNPs-probes aggregation upon the addition of HCl in samples devoid of target DNA. Red color indicates AuNPs-probes dispersion and purplish color indicates aggregated nanoparticle

Various DNA concentrations (32 pg, 640 fg, 320 fg, 32 fg and 16 fg) were subjected for analysis and the threshold of detection respective to each strain was determined (Fig. 1). Ensuing hybridization of probe with the target DNA, the solution was supplemented with HCl, rendering NPs to aggregate if not hybridized with the target DNA.

### Clinical sample analysis

Clinical samples (n = 101) encompassing 25 VL and 76 CL species along with 18 non-*Leishmania* skin wounds were investigated. All samples were collected from southern Iran.The samples were previously analyzed via Giemsa staining of smears of lesions (observed by light microscope), biopsies or blood smears and confirmed whit molecular methods. All VL were *L. infantum* and All CL were *L*. *major*. Samples containing amastigote form of *Leishmania* spp were considered as positive controls. Additionally, 25 blood samples collected from non-endemic regions were considered as negative controls.

## Results

### Characterization of AuNPs and AuNPs-probe conjugate

**Fig. 2:**
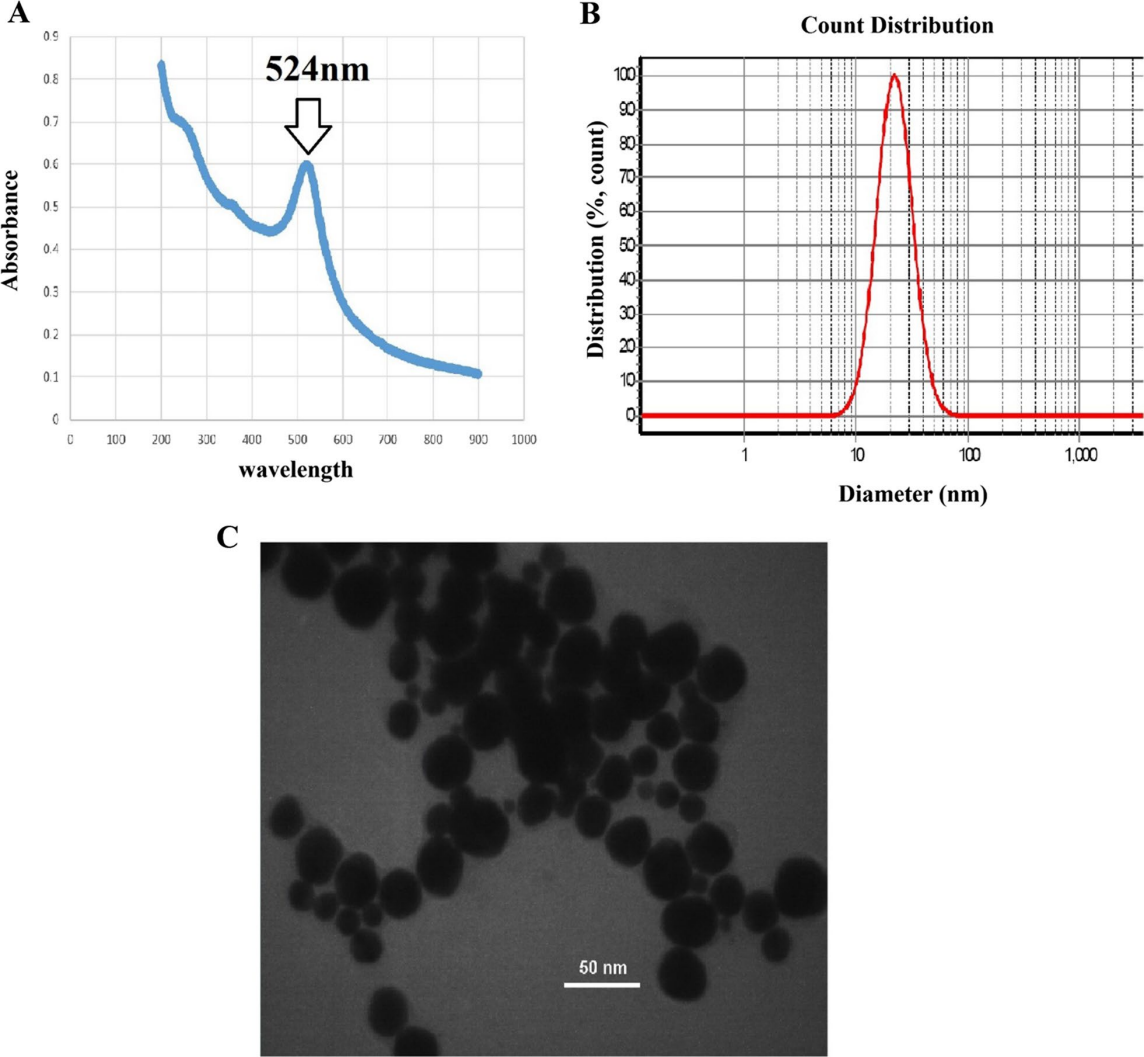
**A** UV–vis spectrum which illustrates the highest absorption wavelength (524 nm) of the NPs, **B** the size distributions of AuNPs via DLS analysis and C: TEM analysis of the NPs size being ~ 20 nm

The single and probe-conjugated AuNPs were subjected to UV–vis spectroscopy, DLS and TEM analysis. The single particles size diameter included 20 ± 5 nm using DLS analysis. The NPs deciphered the highest absorption at 524 nm wavelength (Fig. 2A and B). Moreover, the size of NPs using TEM analysis included ~ 20 nm (Fig. 2C).

**Fig. 3:**
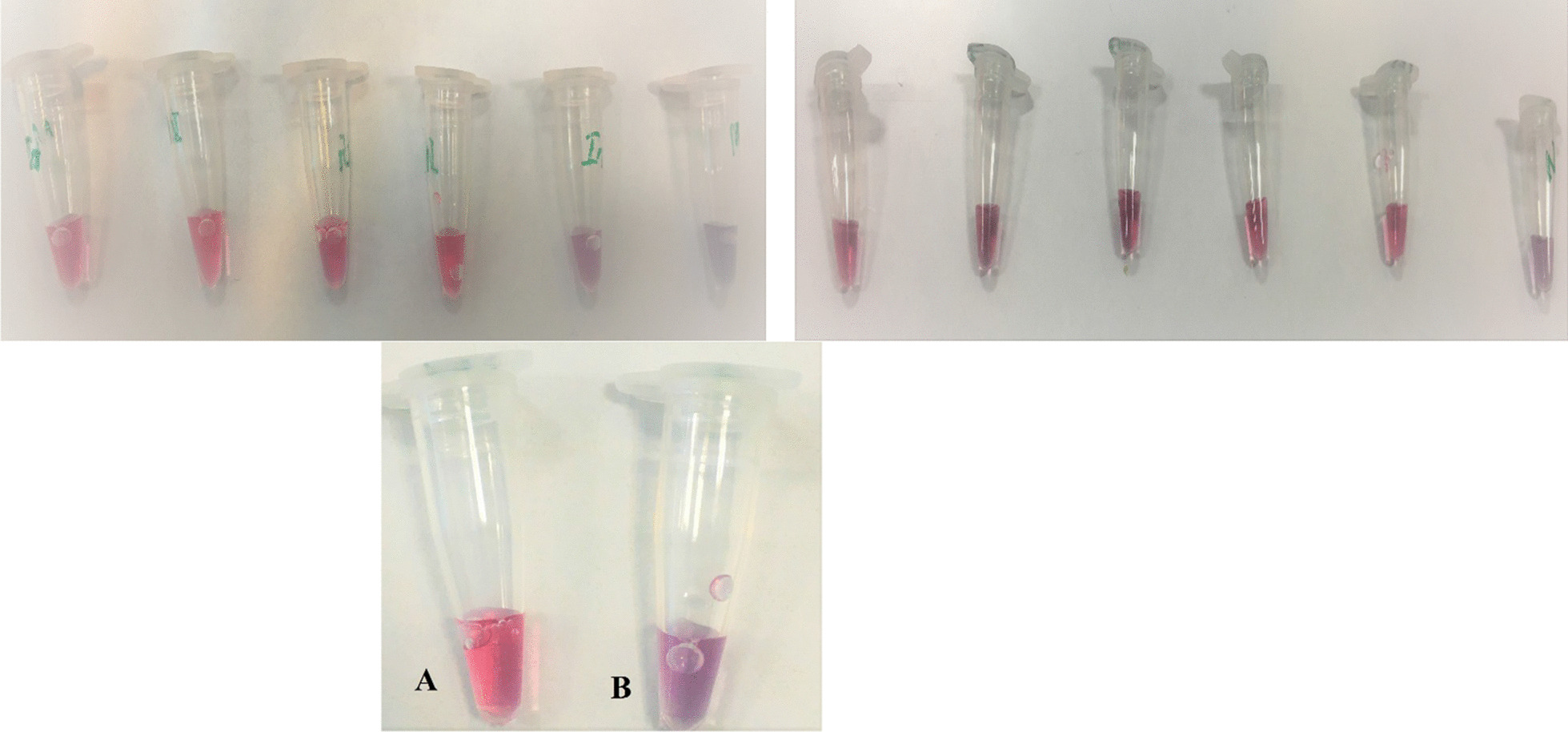
Resistance of conjugated (**A**) and non-conjugated (**B**) NPs aginst MgCl_2_. The red color indicates dispersed nanoparticles whereas the purple color indicates aggregated nanoparticles

The binding of probes with NPs were investigated via resistance analysis in exposure to the MgCl_2_. It was demonstrated that conjugated NPs had a higher resistance toward MgCl_2_ induced aggregation when compared with NPs singly (Fig. 3).

### The threshold of detection

**Fig. 4:**
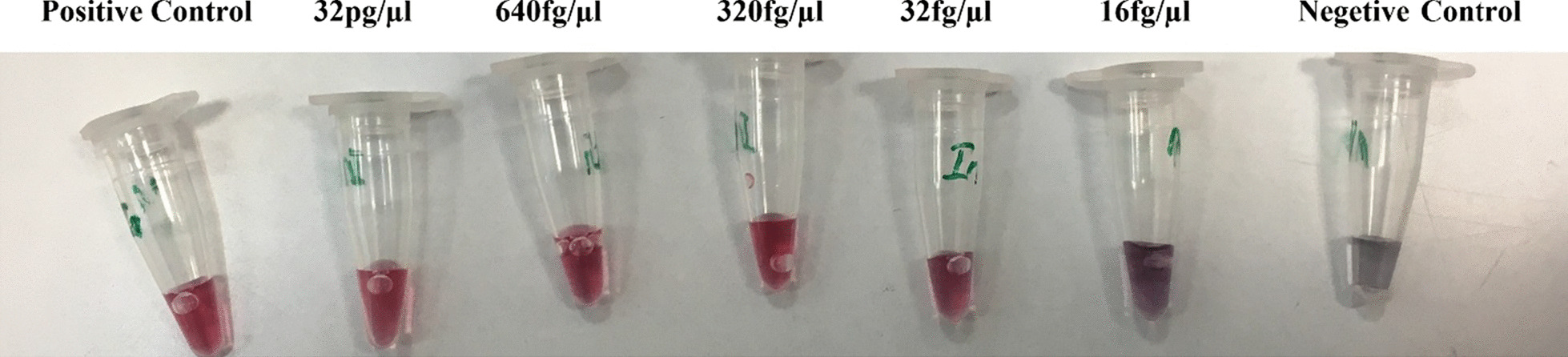
Determining the threshold of detection using serially diluted *Leishmania* DNA. DNA concentrations ranging from 32 to 640 fg/µL were subjected to analysis, where 32 fg/µL was the lowest DNA concentration considered as detection threshold

The threshold of detection was determined through analysis of various concentrations of DNA. The lowest DNA concentration at which the target DNA was detected for all *Leishmania* spp included 32 fg/µL, which was equivalent to 0.3 parasite/µL (Fig. 4).

**Fig. 5:**
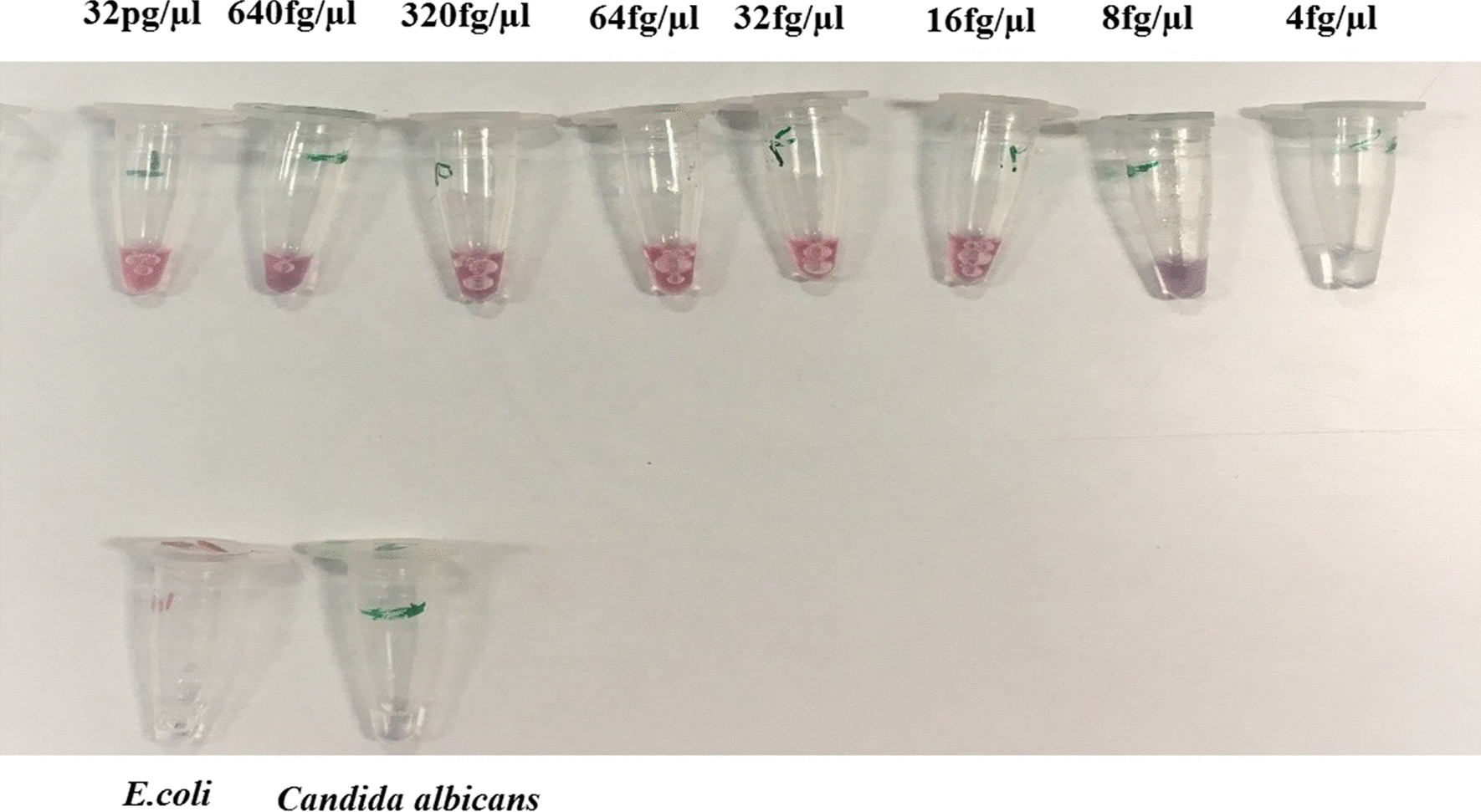
Determining the threshold of detection using the PCR-based agglutination approach. *E. coli* (86.2 ng/µL) and *C. albicans* (43.53 ng/µL) DNA were considered as negative control samples without color change

For the PCR-based agglutination approach, the minimum of threshold was 16 fg/µL (Fig. 5). Hence, lower dilutions of DNA were used in the threshold detection using the PCR-based approach.

The RT-qPCR technique was also performed as the gold standard method of parasite detection and compared to the both assays. The minimum of threshold for the RT-qPCR included 16 fg/µL.

### Hybridization via ultraviolet–visible spectroscopy

**Fig. 6:**
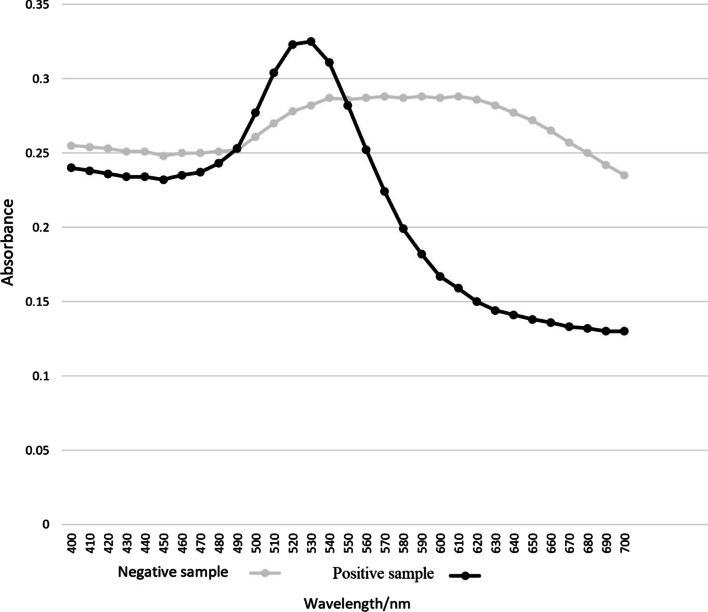
Uv–vis spectroscopy analysis

UV–vis spectra of the samples containing at least 32 fg/µL of parasite DNA showed a maximum absorbance at approximately 528 nm. However, the remaining samples namely blank, negative control and those with DNA concentrations lower than the threshold, reflected the highest absorbance at 524 nm to 610 nm (Fig. 6).

Samples containing target DNA at concentration higher than the threshold demonstrated the highest absorbance at 528 nm rather sharply, while those lacking the target DNA along with negative control and those containing target DNA at a concentration lower than that of threshold had the highest absorbance at roughly 540-610 nm.

### Clinical samples analysis

Majority (75/76) of CL- and VL-positive (24/25) samples were determined positive using AuNPs-probe sensors, and 17 out of 18 negative samples were correctly detected as negative. The specificity and sensitivity of CL included 98% and 94%, respectively, whereas those of VL included 96% and 100%, respectively (Table 1). Accordingly, PCR-based agglutination approach results were identical to that of RT-qPCR; however, the results for conjugated NPs were marginally lower. There was no significant difference between blood and tissue samples in terms of sensitivity and specificity of the test (p > 0.05).

**Table1:** Overall analysis of the clinical samples via RT-qPCR, AuNPs-probe conjugate and PCR-based agglutination approach

Clinical samples	AuNPs-probe conjugate		PCR-based agglutination approach		Real-time PCR	
	** + **	**−**	** + **	**−**	** + **	**−**
Non-*Leishmania* skin wounds (n = 18)	1	17	1	17	1	17
VL (n = 25)	24	1	25	–	25	–
Blood samples from non-Endemic regions (n = 25)	–	25	–	25	–	25
CL (n = 76)	75	1	76	–	76	–
Specificity for VL	100%		100%		100%	
Sensitivity for VL	96%		100%		100%	
Specificity for CL	94%		94%		94%	
Sensitivity for CL	98%		100%		100%	

*VL* visceral leishmaniasis, *CL* cutaneous leishmaniasis

## Discussion

AuNPs-based DNA biosensors coated with single-stranded DNA oligonucleotides via thiol bonds have gained widespread applications and become more significant in the fields of diagnostics, forensics and patient follow-up [19, 20]. Here, we sought to select an oligonucleotide with high conservancy among different species of *Leishmania* to serve as a specific probe with scavenging ability through heterogeneous samples to bind to its target DNA. The oligonucleotide was thoroughly analyzed via bioinformatics tools ensuring that would solely bind to *Leishmania* spp DNA. Conventional diagnostic approaches such as culture, microscopic examination and serology methods are labor-working and have poor sensitivity for the parasite identification. [7, 21, 22]. While immunoassay methods have been employed to diagnose leishmaniasis such as ELISA, the tests have exhibited cross-reactivity with other parasitic agents namely *Trypanosoma* and *Plasmodium* spp., rendering the immunoassay methods rather non-specific. Restriction fragment length polymorphism, PCR and RT-qPCR are further molecular approaches; however, they are cost-intensive as extravagant high-tech facilities are required [5, 18, 19]. Thus, we opted for designing an AuNP-based biosensor to improve drawbacks, as they contain advantages such as easy production and cost-effectiveness. These nanobiosensors do not require elaborate approaches for performance and detection or simple agglomeration is convenient via naked eye. The oligonucleotide attached to the nanoparticles serve as the probe that captures the target DNA of *Leishmania* spp. Since the specificity and sensitivity of the test is mainly dictated by the oligo probe, the ITS2 highly conserved among *Leishmania* species was selected as the sequence of interest, ensuring that the oligo probes would solely bind to the target DNA in particularly in unsuitable conditions. ITS2 oligo probe specificity was high in this study. In terms of sensitivity, the ITS2 probe was capable of binding to minute amount of DNA present in samples (32 fg/µL). Although the selection of oligo probe was highly important in the test′s efficacy, the nanobiosensors also exploit the advantages of AuNPs. Therefor the high specificity and sensitivity along with the extremely low threshold of detection are the cumulative result of ITS2 and AuNPs properties. From manufacturing vantage point, nanoparticle preparation and validating the final products require basic lab equipment, for instance a spectrophotometer, which can be found in every laboratory worldwide. There are, however, some facilities such as a dynamic light scattering machine which may seem less routine; however, in comparison to qPCR etc. they are far more easily to use and frequent among laboratories. Since nanoparticles are stable substances they can be mass-produced and easily distributed to laboratories. In terms of detection, nanoparticle aggregation is an efficient and cost-effective approach without costly materials or equipment requirements. Nanobiosensors could be also used as a point-of-care testing as they are extremely easy to transport and the detection does not require any laboratory equipment. This is particularly important for faraway regions, where medical staff do not have access to laboratory equipment.

ITSs are removed from the primary transcript by snoRNAs and multiple enzymes [23, 24]. Analysis of available bioinformatics databases has revealed different subunits of the rRNAs of *L. infantum* located in different chromosomes and are not arranged in a head-to-tail tandem array. Moreover, the number of rRNA gene sets in *L. donovani* haploid genome is equal to 166. It is estimated that several copies of rRNA gene repeats exist in the diploid genome of different *Leishmania* species, which makes it a good target for analyzing low parasite numbers [25]. Thus in this study, ITS2 conserved sequence was adopted and the sensitivity and specificity of conjugation method was similar to those of PCR and qPCR amplification methods giving rapid results. ITS2 as a specific diagnostic probe, and an optical sensor conjugated to AuNPs was developed. rRNA gene is a proper candidate for designing an oligonucleotide probe for bacteria biosensing due to multiple copies per genome which can improve the assay sensitivity and lower detection limit.

In this study, we developed a simple assay based on a non-cross-linking hybridization method, where the aggregation of AuNPs-probe conjugate was induced by an increasing acid concentration. In *Leishmania* DNA-positive samples, the presence of a complementary target prevented this aggregation and the solution remained red, the solutions of AuNPs and AuNP-probe in *Leishmania* are red with a relatively narrow surface plasmon absorption band at 528 nm in UV–vis spectrum. In contrast, the negative samples and other bacterial DNA, such as *T. gondii, E. coli and C. albicans* contained non-complementary targets which did not prevent AuNPs-probe aggregation, resulting in a color change from red to purple with a corresponding red shift in surface plasmon resonance from 528–580 nm. These results indicated that the designed colorimetric biosensor could discriminate different target sequences effectively and displayed excellent selectivity forms. Currently, such biosensors are used to identify various agents causing infectious diseases [26–28].

The developed *Leishmania* spp AuNP-probe conjugate assay had a detection limit of 32 fg/µL of unamplified *L. major, L. tropica and L. infantum* DNA fragments. Sensitivity of this technique for CL and VL included 98% and 96%, respectively. In other assays developed by Vanani et al. [9] and [16], Andreadou et al. [15], Moradi et al. [8], the detection limits of *Leishmania spp* were 1.2 ng, 0.07 ng, 11.5 ng and 0.07 ng/ μL, respectively. The appropriate limit of quantification in these studies can be considered as a result of several factors involved in the preparation of the desired biosensor, including excellent electrical conductivity, structural properties, different marker and high stability of the AuNPs. A recent study using AuNP-probe and molecular methods outlined detection limits of 10^2^ parasites/mL (0.0147 ng/µL) for the diagnosis of VL among human immunodeficiency virus (HIV) infected patients [29]. This method offered promising insights into the *Leishmania* spp., detection in a reliable and highly specific manner without any cross-reactivity with other similar agents.

## Conclusion

Owing to the accuracy, high sensitivity and specificity and validity of the adopted AuNPs-probe conjugated biosensor designed in this study, its application for the screening of suspicious patients is promising, particularly in lower-developed areas. The ITS2 conserved sequence was adopted and the sensitivity and specificity of conjugation method was similar to those of PCR and RT-qPCR amplification methods which provided rapid results.

## Data Availability

The datasets used and/or analysed during the current study will be available from the corresponding author on reasonable request.
